# The 100 Top-Cited Systematic Reviews/Meta-Analyses on Diabetic Research

**DOI:** 10.1155/2020/5767582

**Published:** 2020-09-11

**Authors:** Yi Yang, Yao Ma, Lingmin Chen, Yuqi Liu, Yonggang Zhang

**Affiliations:** ^1^Department of Periodical Press and National Clinical Research Center for Geriatrics, West China Hospital, Sichuan University, Chengdu, 610041 Sichuan, China; ^2^Department of Clinical Medicine, Gansu University of Traditional Chinese Medicine, Lanzhou 730020, China; ^3^The Center of Gerontology and Geriatrics, West China Hospital, West China School of Medicine, Sichuan University, Chengdu, China; ^4^West China School of Medicine, Sichuan University, Chengdu 610041, China; ^5^Department of Endocrinology and Metabolism, West China Hospital, Sichuan University, Chengdu 610041, China; ^6^Chinese Evidence-Based Medicine Center, West China Hospital, Sichuan University, Chengdu 610041, China

## Abstract

**Objective:**

The objective of this study was to analyze the 100 top-cited systematic reviews/meta-analyses on diabetic research.

**Methods:**

The Science Citation Index Expanded database was searched to identify top-cited studies on diabetic research up to March 4^th^, 2020. Studies were analyzed using the following characteristics: citation number, publication year, country and institution of origin, authorship, topics, and journals.

**Results:**

The 100 top-cited diabetic systematic reviews/meta-analyses were published in 43 different journals, with *Diabetes Care* having the highest numbers (*n* = 17), followed by *The Journal of the American Medical Association* (*n* = 14) and *Lancet* (*n* = 9). The majority of studies are published in the 2000s. The number of citations ranged from 2197 to 301. The highest number of contributions was from the USA, followed by England and Australia. The leading institution was Harvard University. The hot topic was a risk factor (*n* = 33), followed by comorbidity (*n* = 27).

**Conclusions:**

The 100 top-cited systematic reviews/meta-analyses on diabetic research identify impactful authors, journals, institutes, and countries. It will also provide the most important references to evidence-based medicine in diabetes and serve as a guide to the features of a citable paper in this field.

## 1. Introduction

According to the WHO, diabetes had been identified as one of the four major noncommunicable diseases [1–3], and the number of deaths due to diabetes increased by 31.1% between 2006 and 2016 [4, 5]. Currently, about 382 million adults (8.3%) are living with diabetes, and it will be over 592 million by 2035 [4, 5]. As a consequence, many diabetic studies have been published during the past few decades [6–9]. Along with the increasing of the literature in original diabetic articles [10, 11], the systematic reviews/meta-analyses on diabetic research are also increasing. Assessment quality and quantity of literature has become much more important in the scientific area, and bibliometrics analysis is the most important involved method [12–14]. Fixing citation thresholds (100-400 citations) and choosing the top-cited studies (top 25 to 100) from a list [15] were the most common bibliometrics analysis method. Up to now, there have been several top-cited studies on various clinical specialties, including anesthesiology [16], tuberculosis [17], orthopedic surgery [18], gastric cancer [19], and gastroenterology [20]. There were also two such studies about diabetes [6, 7]; however, they did not report about the systematic review/meta-analysis.

Systematic reviews/meta-analyses use systematic methods to collect secondary data, critically appraise research studies, and synthesize studies [11, 21–27]. They are designed to provide a complete, exhaustive summary of current evidence relevant to a research question and are the key to the practice of evidence-based medicine and have always been used in practice guideline [28–30]. Assessment of quality and quantity of diabetic systematic reviews/meta-analyses by bibliometrics analysis should be very important for diabetic research. Analyzing the top-cited systematic reviews/meta-analyses will help us to know the hottest topic and contribute future works in such a field. However, there was no such study. Thus, we performed the current study to assess the 100 top-cited systematic reviews/meta-analyses in diabetic research.

## 2. Methods

### 2.1. Inclusion and Exclusion Criteria

The inclusion criteria were as follows: (a) the study design should be a systematic review or meta-analysis or a systematic review and meta-analysis or a Cochrane review; (b) the study should be on diabetes, for example, if the review analyzed the changes in the blood glucose level in old diabetes mellitus patients, it could be included; if the review analyzed how to detect blood glucose by using blood glucose meters, it should not be included. The exclusion criteria used were (1) abstracts or reviews and (2) study focusing on the diabetes-associated issues, such as investigating the mechanism of antidiabetic drugs or investigating nondiabetic issues.

### 2.2. Identification of the 100 Top-Cited Studies

A retrospective bibliometric analysis was performed on March 4^th^, 2020 to identify studies using the Web of Science Core Collection. The following search strategy was used: “diabetes or diabetic” and “systematic review or meta-analysis”, in combination with “diabetes or diabetic” and “PUBLICATION NAME: (Cochrane database of systematic reviews)”. The search results were subsequently ranked according to the number of citations. Two authors screened the titles and abstracts and identified the 100 top-cited studies on diabetic research. In cases of discrepancy, the consensus was achieved with the help of a third independent author.

### 2.3. Analysis of the 100 Top-Cited Studies

The following information was extracted from each article: authorship, source journal, year of publication, geographic origin, scientific research institution, number of citations, and research topics. If the author belonged to different institutions, the first institution for the author was used for data analysis. If there was only one author, the first author was simultaneously recognised as the corresponding author. Impact factors (IF) from the Journal Citation Report (JCR) reported published in 2019. If the journal has changed its name, the IF was identified based on its current name. Two authors independently identified the research topics as six topics, including drug therapy, complication, comorbidity, related treatment, risk factors, and others. The following definitions were used: drug therapy studies were defined if the study focused on drug therapy of any types of diabetes; complication studies were identified if the study focused on diabetic complications; comorbidity studies were defined if the study focused on comorbidities related to diabetes; related treatment studies were defined if the study focused on associated treatments, such as exercise and self-management; risk factor studies were identified if the study focused on investigating potential risk factors for diabetes; if a study cannot be clarified into the above 5 topics, it will be defined as the other topics. To avoid potential studies which could be identified into two topics, each study was identified in the following orders, drug therapy, complication, comorbidity, related treatment, risk factors, and others. In cases of discrepancy, the consensus was achieved with the help of a third independent author. VOSviewer 1.6.6 (http://www.vosviewer.com/, Leiden University Centre for Science and Technology Studies) was used to analyze the cocitation of the top 100 studies.

## 3. Results

### 3.1. The Main Characteristics of the Included Studies

Supplement table 1 shows the characteristics of the 100 top-cited studies in descending order. The citation times of these studies varied from 2197 to 301, with a total citation time of 60180. The most cited study with 2197 citation times was a meta-analysis of the prevalence of comorbid depression in adults with diabetes, which was published in *Diabetes Care* [31]. The second study was a meta-analysis named “Diabetes mellitus, fasting blood glucose concentration, and risk of vascular disease: a collaborative meta-analysis of 102 prospective studies,” which was published in *Lancet* and cited 1726 times [32]. The third study was a meta-analysis of weight and type 2 diabetes after bariatric surgery, which was published in the *American Journal of Medicine* and cited 1540 times [33].

### 3.2. Distribution of Authors

The authors published at least two studies as first authors or corresponding authors are shown in Table 1. Two authors published three studies as the first author or corresponding author, including Rob M. van Dam and Anastassios G. Pittas. For the first authors, all three authors were experts in the field of public health. While for the corresponding authors, five authors were experts in the field of public health, only 1 was an expert in the field of diabetes.

### 3.3. Distribution of Countries

The 100 top-cited studies on diabetes were from 19 countries, including USA, England, Australia, Netherlands, Canada, Sweden, Germany, Scotland, China, Brazil, Switzerland, South Korea, Denmark, France, Greece, Iran, Italy, Japan, New Zealand, and North Ireland. The countries produced the most significant number of studies were the USA (*n* = 40), England (*n* = 20), and Netherlands (*n* = 6). The studies produced most citations were from the USA, with 26450 citation times, followed by England with 13305 citation times. The country with the most average citation was Scotland with 1108 citation times, followed by Denmark with 963 citation times (Table 2).

### 3.4. Distribution of Institutions

A total of 16 institutions with more than two studies were included (Table 3). The institutions with the most of studies were Harvard University in the USA (*n* = 11), followed by the University of Cambridge (n  =  3) and University of Leicester (*n* = 3) in England, University of Sydney (*n* = 3) in Australia, Johns Hopkins University (*n* = 3), University of Michigan (*n* = 3) and Tufts University (*n* = 3) in the USA, and Karolinska Institutet (*n* = 3) in Sweden.

### 3.5. Distribution of Published Years

Year's distribution of the 100 top-cited studies is shown in Table 4. These studies were published from 1996 to 2015. The year with most studies was 2007 with 18 studies, followed by 2008 with 11 studies. The year with most citations was 2007 with 10488 citations, followed by 2008 with 6675 citations. The year with most average citation was 2001 with 1153 citations, followed by 2002 with 843 citations.

### 3.6. Distribution of Published Journals

The 100 studies were published in 42 journals (Table 5). The journal with the largest number of articles cited was *Diabetes Care* (*n* = 17), followed by *JAMA* (*n* = 14) and *Lancet* (*n* = 9).

### 3.7. Distribution of Research Topics

Topic distribution of the 100 top-cited studies is shown in Table 6. The hottest topic was the risk factor (*n* = 29); the most average citation was drug therapy.

### 3.8. Cocitations

The cocitation of the 100 top-cited studies is shown in Supplement Figure 1. The most frequent cocitation study was about quantifying heterogeneity in a meta-analysis (*n* = 15) published by Higgins JP in 2002. The most frequent cocitation source was *Diabetes Care* (*n* = 239). Jürgen Rehm from the University of Toronto was the most frequent cocitation author (*n* = 36).

## 4. Discussion

The results of our study showed that the 100 top-cited studies were cited 2197 to 301 times, which is much less than the previous studies for all diabetic researches (ranged from 10292 to 1121). When compared with tuberculosis, the number is much higher than the previous studies about tuberculosis studies [34]; the reason may be that the number of researchers in the diabetic field may more than that in the tuberculosis field.

The years in which most of the top-cited diabetic studies published are the 2000s. In all, most of the studies were published between 2005 to 2012, and 18 were published in 2007, which accounted most in the years, which suggested that it might take about ten years for systematic review citation to peak, which was consistent with results from tuberculosis [34].

Our study found that most top-cited studies were from the USA, followed by England and Canada. The results were in line with the origin of the 100 most frequently cited articles in many other fields. The USA leads the world in medical researches, given its large number of researchers and generous research funding [34, 35]. Most studies were written by researchers in the USA, England, and Canada. Thus, most of the top-cited studies were from these countries.

The results from our analysis indicated that the most top-cited studies were published in journals related to endocrinology and metabolism, such as *Diabetes Care*, *Diabetologia*, and *Diabetic Medicine*. Comprehensive medical periodicals have also published top-cited studies, such as *JAMA*, *Lancet*, *BMJ*, and *Annals of Internal Medicine*. We have to mention some journals in the field of cancer, public health, and cardiology, such as the *American Journal of Epidemiology*, *Cancer Epidemiology Biomarkers & Prevention*, *American Heart Journal*, and *British Journal of Cancer*. Diabetes was studied as a risk factor in these studies [36–39]. This may suggest the editors and authors to choose research topics of studies in diabetes in the future [40].

It is very interesting that the citation of the risk factor topic got the highest total citations than the other topics. The reason might be that the risk factor studies attracted more attention from other disciplines except for endocrinology. Among the 100 studies, about 1/3 studies were about the comorbidity, and this would help journals to invite or accept manuscripts.

The most popular topics might be different from the hot topics on the Internet [41], and we needed to measure the number and nature of online attention around the research results. At present, altmetric attention scores, which were calculated using different weight values of different data resources, including Twitter, Facebook, and Google+, were usually used to assess the impact and contribution in many fields. A significant positive correlation between altmetric score and standardized citation might be found in some fields. However, we should also know that bibliometric and altmetric analyses provided important but different perspectives about study impact.

Some limitations of this study should be noted. First, this is a cross-sectional study design with a single time point. The rankings identified might change if the study was replicated in the future. Second, with the increasing launched new journals and published new papers, the papers in recent years might get more citations. Third, the citation counts differ according to the citation database under study. Although the Web of Science database was widely considered as the gold standard used in the top-cited analysis, however, we should not ignore the Google Scholar or Scopus databases. Fourth, due to the time limit of the citation index, some new studies could not be included in this study, and older manuscripts were more likely to be cited by newer manuscripts. Therefore, in future studies, we could use the citation rate index, altmetrics, or PlumX to evaluate the impact of research in this field to eliminate such interference.

In conclusion, we identified the 100 top-cited systematic reviews/meta-analyses on diabetic research. They identified the impactful authors, journals, institutes, and countries and also analyzed the most popular articles and topics in the field. It will also provide the most important references related to evidence-based medicine in diabetes and serve as a guide to the features of a citable paper in this field.

## Figures and Tables

**Table 1:** Authors with more than one study as first or corresponding authors included in the 100 top-cited systematic reviews/meta-analyses for diabetes.

	Name	Number of study	Affiliation	Professional
First author	Rachel Huxley	2	University of Sydney	Public health
	Susan L. Norris	2	Centers for Disease Control and Prevention	Public health
	Larsson C. Susanna	2	Karolinska Institutet	Public health

Corresponding author	Huxley Rachel	2	University of Sydney	Public health
	Susan L. Norris	2	Centers for Disease Control and Prevention	Public health
	Anastassios G. Pittas	3	Tufts-New England Medical Center	Diabetes
	Larsson C. Susanna	2	Karolinska Institutet	Public health
	Rob M. van Dam	3	VU University Medical Center	Public health
	Hu. Frank B	2	Harvard University	Public health

**Table 2:** Country of origin of the 100 top-cited systematic reviews/meta-analyses for diabetes (based on the country of the corresponding author).

Ranking	Country	Number of study	Total citation	Highest times of citation	Lowest times of citation	Average citation
1	USA	40	26450	2197	301	661
2	England	20	13305	1726	311	665
3	Netherlands	6	3212	1052	350	535
3	Australia	6	2470	885	312	542
5	Canada	4	2048	960	344	512
5	Sweden	4	1788	631	304	447
7	Germany	3	1116	434	324	372
8	Scotland	2	2215	1393	822	1108
8	Brazil	2	873	531	342	437
8	China	2	674	359	315	337
8	Switzerland	2	971	667	304	486
12	South Korea	1	365	365	365	365
12	Denmark	1	963	963	963	963
12	France	1	372	372	372	372
12	Greece	1	412	412	412	412
12	Iran	1	617	617	617	617
12	Italy	1	523	523	523	523
12	Japan	1	329	329	329	329
12	New Zealand	1	387	387	387	387
12	North Ireland	1	307	307	307	307

**Table 3:** Institutions with at least 2 studies based on the institution of the corresponding authors included in 100 top-cited systematic reviews/meta-analyses for diabetes.

Institution	Country	Number of study	Total citation	Highest citation	Lowest citation	Average citation
Harvard University	USA	11	5029	669	304	457
University of Cambridge	England	3	2874	1726	324	958
University of Sydney	Australia	3	1975	885	401	652
University of Leicester	England	3	1995	725	621	665
Johns Hopkins University	USA	3	1913	924	474	638
University of Michigan	USA	3	2449	1189	529	816
Tufts University	USA	3	2337	1132	463	779
Karolinska Institutet	Sweden	3	1157	525	304	356
Washington University	USA	2	3192	2197	995	1596
Free University of Amsterdam	Netherlands	2	927	534	393	464
Centers for Disease Control and Prevention	USA	2	2087	1139	952	1043
University of Melbourne	Australia	2	768	456	312	384
University of Glasgow	Scotland	2	2215	1393	822	1108
University of Minnesota	USA	2	2003	1540	463	1002
University of Oxford	England	2	1638	1315	323	819

**Table 4:** Distribution by year of publication of the 100 top-cited systematic reviews/meta-analyses for diabetes.

Year	Number of study	Total citation	Highest citation	Lowest citation	Average citation
2015	2	794	397	323	360
2014	1	529	529	529	529
2013	2	727	412	315	364
2012	7	2870	725	317	410
2011	9	3787	822	304	421
2010	9	6668	1726	314	741
2009	8	6437	1540	311	793
2008	11	6675	1315	307	607
2007	18	10448	1132	304	580
2006	10	5796	1052	304	579
2005	8	3671	671	301	459
2004	3	2090	924	434	697
2003	3	1092	454	303	364
2002	3	2530	1189	389	843
2001	5	5767	2197	480	1153
1996	1	463	463	463	463

**Table 5:** Journals in which the 100 top-cited systematic reviews/meta-analyses for diabetes were published.

Ranking	Name of journal	Number of study	Impact factor^#^
1	Diabetes Care	17	15.27
2	JAMA-Journal of the American Medical Association	14	51.273
3	Lancet	9	59.102
4	Nature Genetics	5	25.455
5	Diabetologia	5	7.113
6	BMJ-British Medical Journal	4	27.604
7	Annals of Internal Medicine	4	19.315
8	Diabetic Medicine	3	3.107
9	American Journal of Clinical Nutrition	2	6.568
10	American Journal of Epidemiology	2	4.473
11	Cochrane Database of Systemic Reviews	2	7.755
12	Journal of the American College of Cardiology	2	18.639
13	PLOS Medicine	2	11.048
14	American Heart Journal	1	4.023
15	American Journal of Medicine	1	4.76
16	Archives of Disease in Childhood	1	3.158
17	Archives of Internal Medicine	1	20.768
18	Biological Psychiatry	1	11.501
19	BMC Medicine	1	8.285
20	British Journal of Cancer	1	5.416
21	Canadian Medical Association Journal	1	6.938
22	Cancer Epidemiology, Biomarkers & Prevention	1	5.057
23	Cancer Prevention Research	1	3.866
24	Circulation	1	23.054
25	Clinical Gastroenterology and Hepatology	1	7.958
26	Epidemiologic Reviews	1	6.455
27	European Heart Journal	1	23.239
28	European Journal of Cancer	1	6.68
29	European Journal of Clinical Nutrition	1	3.114
30	Human Reproduction Update	1	12.878
31	Internal Medicine Journal	1	1.767
32	International Journal of Cancer	1	4.982
33	International Journal of Epidemiology	1	7.339
34	Journal of Affective Disorders	1	4.084
35	Journal of Clinical Endocrinology and Metabolism	1	5.605
36	Journal of the National Cancer Institute	1	10.211
37	Lancet Neurology	1	28.755
38	Nutrition Research Reviews	1	5.595
39	Obesity Reviews	1	8.192
40	Osteoporosis International	1	3.819
41	PLOS One	1	2.776
42	Psychosomatic Medicine	1	3.937

#: from the Journal Citation Report in 2016; ∗: QJM-an international journal of medicine; &: JAMA internal medicine; JAMA: The Journal of the American Medical Association; BMJ: British Medical Journal.

**Table 6:** Distribution by topics of the 100 top-cited systematic reviews/meta-analyses for diabetes.

Topic	Number of study	Total citation	Average citation
Drug therapy	22	13777	626
Complication	9	1801	300
Comorbidity	25	14660	586
Related treatment	13	7840	603
Risk factor	29	16697	576
Other	2	973	587

## Data Availability

The original data used to support the findings of this study are included within the article.

## Abbreviations

JCR: Journal Citation Report
IF: Impact factor
JAMA: The Journal of the American Medical Association
BMJ: British Medical Journal.

## Supplementary Materials

Supplementary 1 Supplement Figure 1: the cocitation between the included studies.

Supplementary 2 Supplement Table 1: the 100 top-cited systematic reviews/meta-analyses in diabetes research.

## References

[1] Chaker L., Falla A., van der Lee S. J. (2015). The global impact of non-communicable diseases on macro-economic productivity: a systematic review. *European Journal of Epidemiology*.

[2] Jiang T. N., Li Y. F., Huo L. L. (2019). Association between serum uric acid and large-nerve fiber dysfunction in type 2 diabetes: a cross-sectional study. *Chinese Medical Journal*.

[3] Chen J., Guo H. J., Qiu S. H. (2018). Identification of newly diagnosed diabetes and prediabetes using fasting plasma glucose and urinary glucose in a chinese population: a multicenter cross-sectional study. *Chinese Medical Journal*.

[4] Lovic D., Piperidou A., Zografou I., Grassos H., Pittaras A., Manolis A. (2020). The growing epidemic of diabetes mellitus. *Current Vascular Pharmacology*.

[5] GBD 2015 Eastern Mediterranean Region Diabetes and Chronic Kidney Disease Collaborators (2018). Diabetes mellitus and chronic kidney disease in the Eastern Mediterranean Region: findings from the Global Burden of Disease 2015 study. *International Journal of Public Health*.

[6] Shuaib W., Costa J. L. (2015). Anatomy of success: 100 most cited articles in diabetes research. *Therapeutic Advances in Endocrinology and Metabolism*.

[7] Zhao X., Guo L., Lin Y. (2016). The top 100 most cited scientific reports focused on diabetes research. *Acta Diabetologica*.

[8] Gou B. H., Guan H. M., Bi Y. X., Ding B. J. (2019). Gestational diabetes: weight gain during pregnancy and its relationship to pregnancy outcomes. *Chinese Medical Journal*.

[9] Jiang T. N., Li Y. F., Huo L. L. (2019). Association between serum uric acid and large-nerve fiber dysfunction in type 2 diabetes. *Chinese Medical Journal*.

[10] Zhang C., Ou Q., Gu Y. (2019). Circulating tissue factor-positive procoagulant microparticles in patients with type 1 diabetes. *Diabetes, Metabolic Syndrome and Obesity: Targets and Therapy*.

[11] Zhang M., Hu M. L., Huang J. J., Xia S. S., Yang Y., Dong K. (2019). Association of leukocyte telomere length with non-alcoholic fatty liver disease in patients with type 2 diabetes. *Chinese Medical Journal*.

[12] Mohammed M. F., Chahal T., Gong B. (2017). Trends in CT colonography: bibliometric analysis of the 100 most-cited articles. *The British Journal of Radiology*.

[13] Zhang Y., Quan L., Du L. (2020). The 100 top-cited articles in main allergy journals: a bibliometric analysis. *Iranian Journal of Allergy, Asthma and Immunology*.

[14] Zhang Y., Quan L., Du L. (2019). The 100 top-cited studies in cancer immunotherapy. *Artificial Cells, Nanomedicine, and Biotechnology*.

[15] Chhapola V., Tiwari S., Deepthi B., Kanwal S. K. (2018). Citation classics in pediatrics: a bibliometric analysis. *World Journal of Pediatrics*.

[16] Chen S. Y., Wei L. F., Ho C. M. (2017). Trend of academic publication activity in anesthesiology: a 2-decade bibliographic perspective. *Asian Journal of Anesthesiology*.

[17] Chen L. M., Liu Y. Q., Shen J. N. (2015). The 100 top-cited tuberculosis research studies. *The International Journal of Tuberculosis and Lung Disease*.

[18] Huo Y. Q., Pan X. H., Li Q. B. (2015). Fifty top-cited classic papers in orthopedic elbow surgery: a bibliometric analysis. *International Journal of Surgery*.

[19] Powell A. G., Hughes D. L., Wheat J. R., Lewis W. G. (2016). The 100 most influential manuscripts in gastric cancer: a bibliometric analysis. *International Journal of Surgery*.

[20] Hu S. K., Huang J., Hong W. D., Du X. J., Jin R., Lin T. S. (2017). The 50 most-cited articles in gastroenterology and hepatology from mainland China. *Pakistan Journal of Medical Sciences*.

[21] Jin C., Deng X., Li Y., He W., Yang X., Liu J. (2018). Lymph node ratio is an independent prognostic factor for rectal cancer after neoadjuvant therapy: a meta-analysis. *Journal of Evidence-Based Medicine*.

[22] Zhang J., Zhao T., Xu C., Yu H. (2018). Four polymorphisms in the IL-22 gene and the risk of cancer: a meta-analysis. *Journal of Evidence-Based Medicine*.

[23] Zhang H. M., Shi Y. X., Sun L. Y., Zhu Z. J. (2019). Hepatocellular carcinoma recurrence in living and deceased donor liver transplantation: a systematic review and meta-analysis. *Chinese Medical Journal*.

[24] Yuan L., Zeng Y., Chen Z. Q. (2019). Efficacy and safety of antifibrinolytic agents in spinal surgery: a network meta-analysis. *Chinese Medical Journal*.

[25] Mekuria A. N., Ayele Y., Tola A., Mishore K. M. (2019). Monotherapy with metformin versus sulfonylureas and risk of cancer in type 2 diabetic patients: a systematic review and meta-analysis. *Journal of Diabetes Research*.

[26] Ida S., Kaneko R., Imataka K., Murata K. (2019). Association between sarcopenia and renal function in patients with diabetes: a systematic review and meta-analysis. *Journal of Diabetes Research*.

[27] Guo L., Ma J., Tang J., Hu D., Zhang W., Zhao X. (2019). Comparative efficacy and safety of metformin, glyburide, and insulin in treating gestational diabetes mellitus: a meta-analysis. *Journal of Diabetes Research*.

[28] Sun H., Li Y., Su Y. (2019). Efficacy and safety of anti-EGFR monoclonal antibodies combined with different chemotherapy regimens in patients with RAS wild-type metastatic colorectal cancer: a meta-analysis. *Journal of Evidence-Based Medicine*.

[29] Duffles L. F., Hermont A. P., Abreu L. G., Pordeus I. A., Silva T. A. (2019). Association between obesity and adipokines levels in saliva and gingival crevicular fluid: a systematic review and meta-analysis. *Journal of Evidence-Based Medicine*.

[30] Chokesuwattanaskul R., Bathini T., Thongprayoon C. (2018). Atrial fibrillation following heart transplantation: a systematic review and meta-analysis of observational studies. *Journal of Evidence-Based Medicine*.

[31] Anderson R. J., Freedland K. E., Clouse R. E., Lustman P. J. (2001). The prevalence of comorbid depression in adults with diabetes: a meta-analysis. *Diabetes Care*.

[32] Sattar N., Preiss D., Murray H. M. (2010). Statins and risk of incident diabetes: a collaborative meta-analysis of randomised statin trials. *The Lancet*.

[33] Norris S. L., Engelgau M. M., Venkat Narayan K. M. (2001). Effectiveness of self-management training in type 2 diabetes: a systematic review of randomized controlled trials. *Diabetes Care*.

[34] Zhang Y., Huang J., Du L. (2017). The top-cited systematic reviews/meta-analyses in tuberculosis research. *Medicine*.

[35] Sweileh W. M., al-Jabi S. W., Zyoud S.'. H., Sawalha A. F. (2018). Bibliometric analysis of literature in pharmacy education: 2000-2016. *The International Journal of Pharmacy Practice*.

[36] Larsson S. C., Orsini N., Wolk A. (2005). Diabetes mellitus and risk of colorectal cancer: a meta-analysis. *Journal of the National Cancer Institute*.

[37] Janghorbani M., Van Dam R. M., Willett W. C., Hu F. B. (2007). Systematic review of type 1 and type 2 diabetes mellitus and risk of fracture. *American Journal of Epidemiology*.

[38] Stettler C., Allemann S., Jüni P. (2006). Glycemic control and macrovascular disease in types 1 and 2 diabetes mellitus: meta-analysis of randomized trials. *American Heart Journal*.

[39] Kasper J. S., Giovannucci E. (2006). A meta-analysis of diabetes mellitus and the risk of prostate cancer. *Cancer Epidemiology, Biomarkers & Prevention*.

[40] Puckrin R., Saltiel M. P., Reynier P., Azoulay L., Yu O. H. Y., Filion K. B. (2018). SGLT-2 inhibitors and the risk of infections: a systematic review and meta-analysis of randomized controlled trials. *Acta Diabetologica*.

[41] Shen Y., Wang F., Zhang X. (2018). Effectiveness of internet-based interventions on glycemic control in patients with type 2 diabetes: meta-analysis of randomized controlled trials. *Journal of Medical Internet Research*.

