# Supplementary material for: The 100 Top-Cited Systematic Reviews/Meta-Analyses on Diabetic Research
Source: J Diabetes Res. 2020 Sep 11;2020:5767582. doi: 10.1155/2020/5767582 (PMC7509559; doi:10.1155/2020/5767582)
Supplement: Supplementary 1 — Supplement Figure 1: the cocitation between the included studies. [file 5767582.f1.docx]

### Supplement Figure 1. The co-citation between the included studies

a. The most frequently co-citation reference

Studies by Higgins JP from British Medical Journal, Higgins JP from Statistics in Medicine and Egger M from British Medical Journal were the most frequently co-citation reference.

**
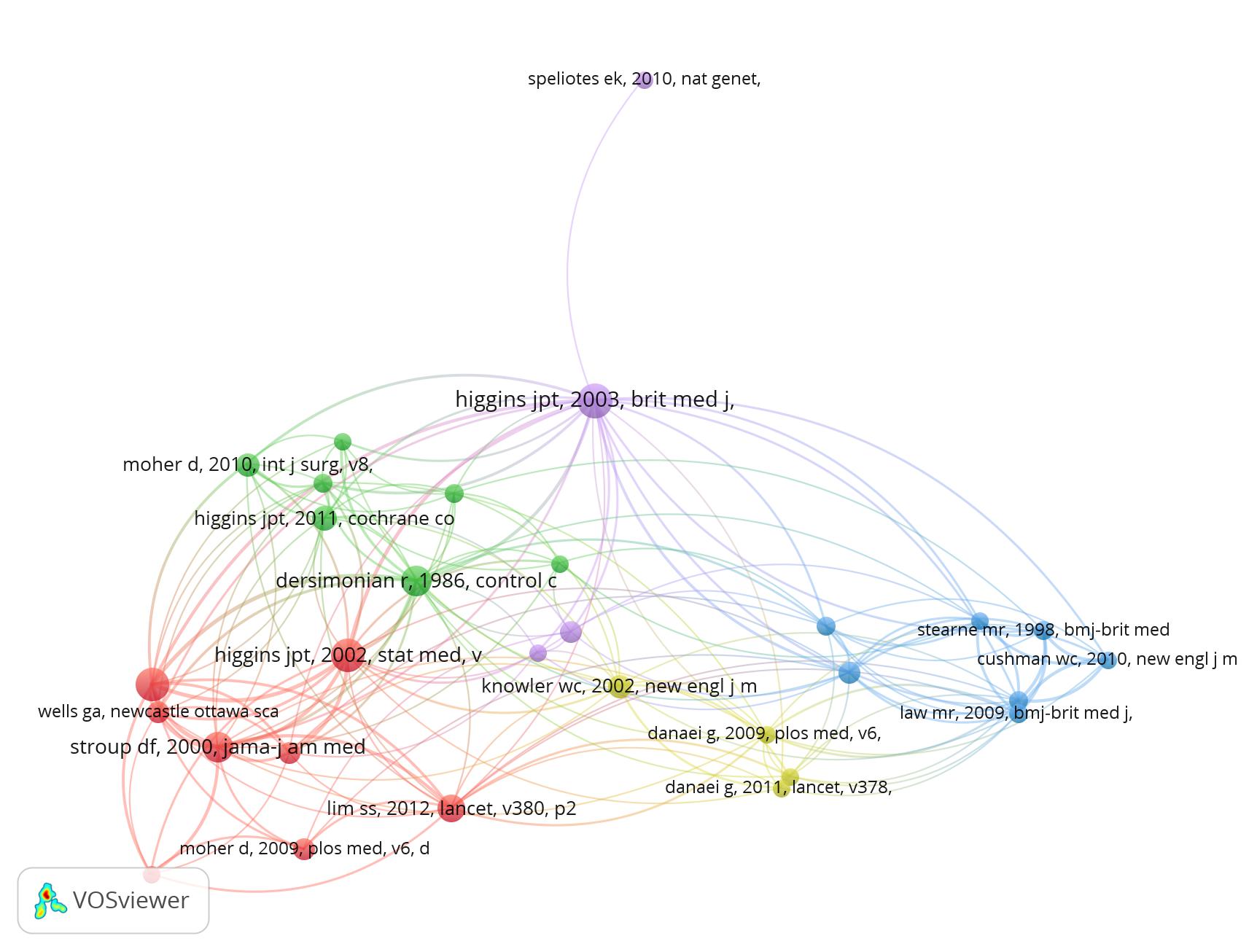
**

b. The most frequently co-citation source

Studies from Diabetes Care, Lancet and New England Journal of medicine were the most frequently co-citation source.

**
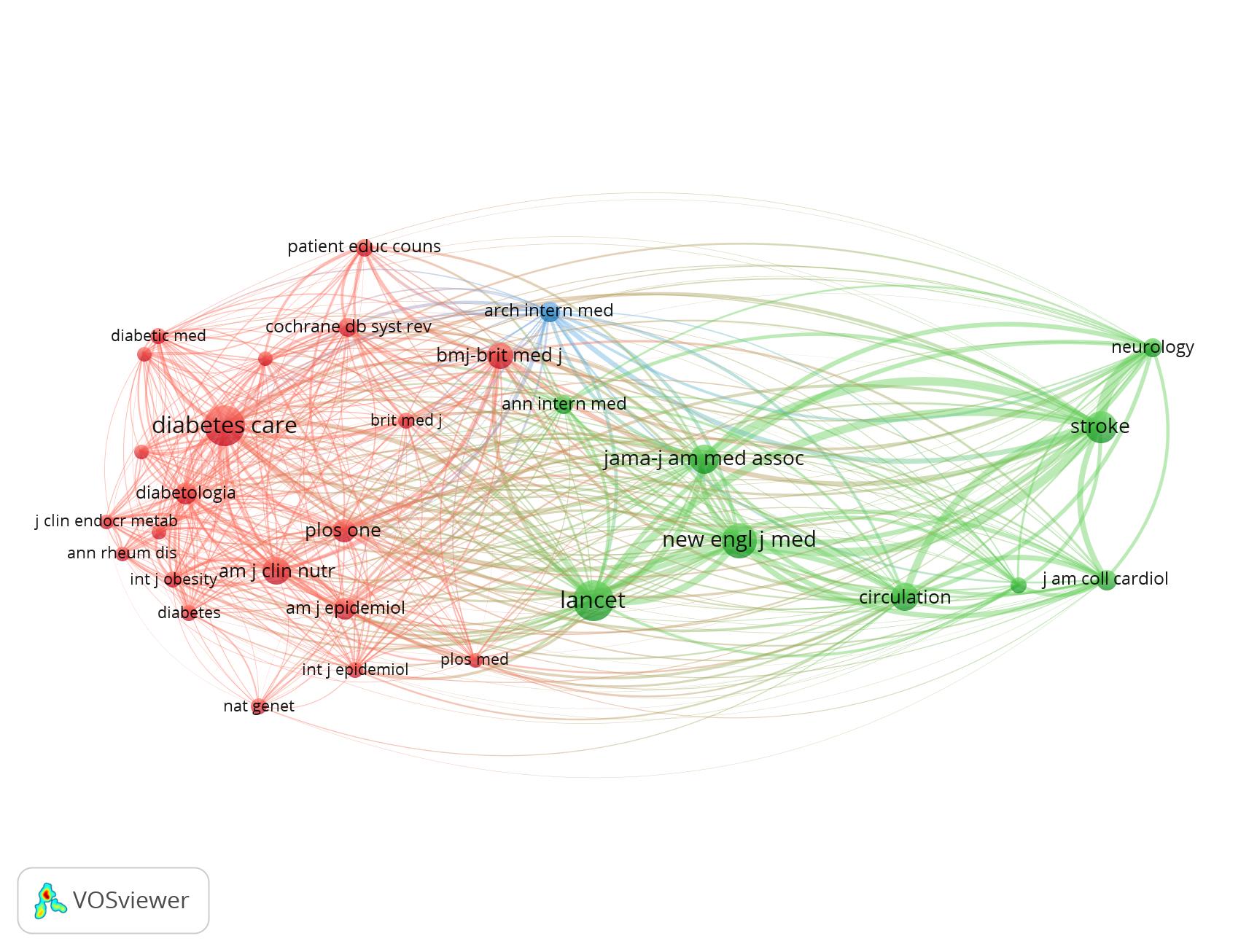
**

c. the most frequently co-citation author

Studies by Higgins JP, Rehm J and WHO are the most frequently co-citation author.

**
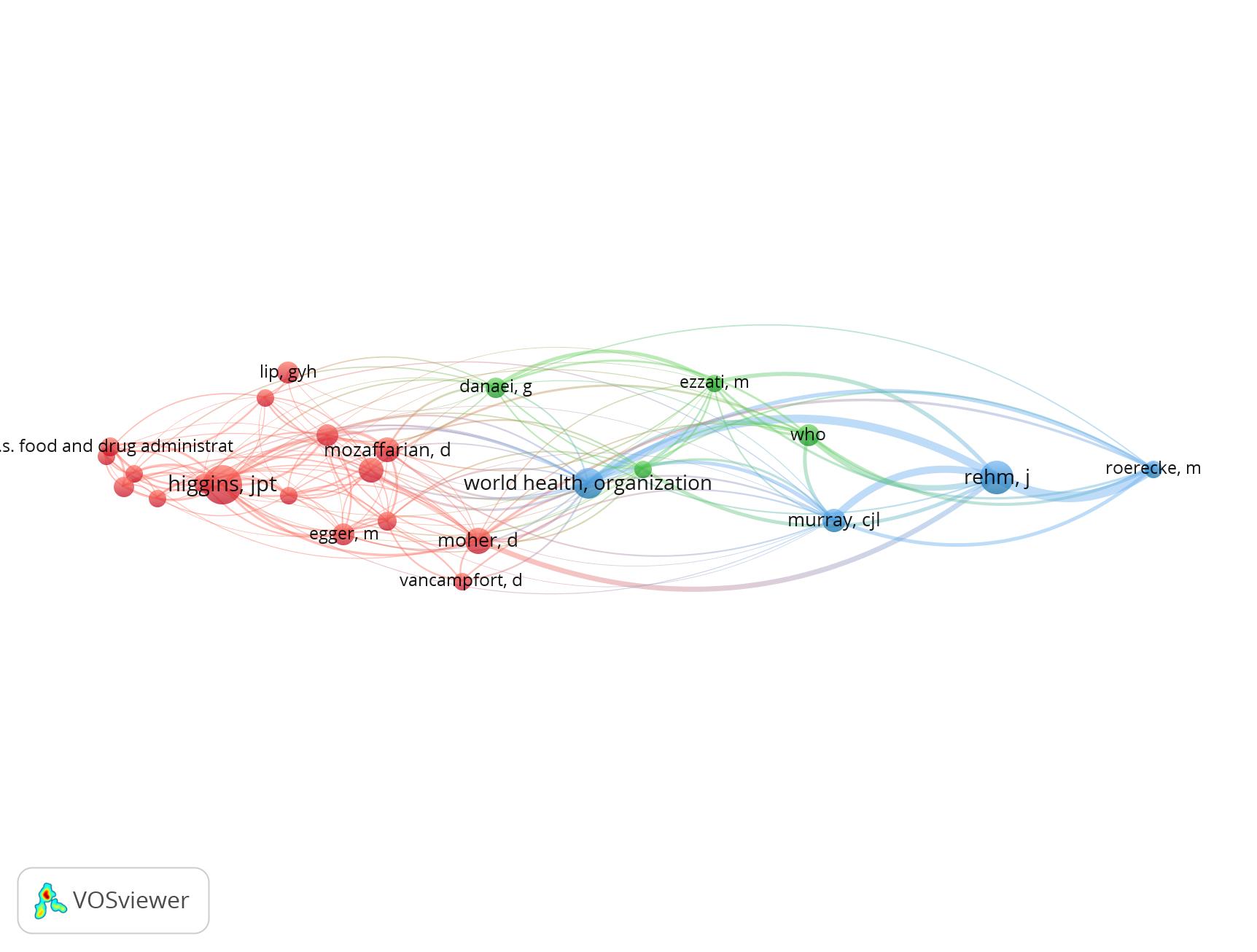
**
