# Supplementary material for: The 100 Top-Cited Systematic Reviews/Meta-Analyses on Diabetic Research
Source: J Diabetes Res. 2020 Sep 11;2020:5767582. doi: 10.1155/2020/5767582 (PMC7509559; doi:10.1155/2020/5767582)
Supplement: Supplementary 2 — Supplement Table 1: the 100 top-cited systematic reviews/meta-analyses in diabetes research. [file 5767582.f2.docx]

**Supplement Table 1 The 100 top-cited systematic reviews/meta-analyses in diabetes research**

| Ranking | Study | Citation times |
| --- | --- | --- |
| 1 | Anderson RJ, Freedland KE, Clouse RE. The prevalence of comorbid depression in adults with diabetes: a meta-analysis. Diabetes Care. 2001;24(6):1069–1078. | 2197 |
| 2 | Sarwar N, Gao P, et al. Diabetes mellitus, fasting blood glucose concentration, and risk of vascular disease: a collaborative meta-analysis of 102 prospective studies. Lancet. 2010;375(9733):2215–2222. | 1726 |
| 3 | Buchwald H, Estok R, Fahrbach K, et al. Weight and type 2 diabetes after bariatric surgery: systematic review and meta-analysis. Am J Med. 2009;122(3):248–256.e5. | 1540 |
| 4 | Sattar N, Preiss D, Murray HM, et al. Statins and risk of incident diabetes: a collaborative meta-analysis of randomized statin trials. Lancet. 2010;375(9716):735–742. | 1393 |
| 5 | Bellamy L, Casas JP, Hingorani AD, Williams D. Type 2 diabetes mellitus after gestational diabetes: a systematic review and meta-analysis. Lancet. 2009;373(9677):1773–1779. | 1369 |
| 6 | Zeggini E, Scott LJ, Saxena R, et al. Meta-analysis of genome-wide association data and large-scale replication identifies additional susceptibility loci for type 2 diabetes. Nat Genet. 2008;40(5):638–645. | 1315 |
| 7 | Kim C, Newton KM, Knopp RH. Gestational diabetes and the incidence of type 2 diabetes: a systematic review. Diabetes Care. 2002;25(10):1862–1868. | 1189 |
| 8 | Cholesterol Treatment Trialists' (CTT) Collaborators, Kearney PM, Blackwell L, et al. Efficacy of cholesterol-lowering therapy in 18,686 people with diabetes in 14 randomised trials of statins: a meta-analysis. Lancet. 2008;371(9607):117–125. | 1164 |
| 9 | Norris SL, Engelgau MM, Narayan KM. Effectiveness of self-management training in type 2 diabetes: a systematic review of randomized controlled trials. Diabetes Care. 2001;24(3):561–587. | 1135 |
| 10 | Pittas AG, Lau J, Hu FB, Dawson-Hughes B. The role of vitamin D and calcium in type 2 diabetes. A systematic review and meta-analysis. J Clin Endocrinol Metab. 2007;92(6):2017–2029. | 1132 |
| 11 | Biessels GJ, Staekenborg S, Brunner E. Risk of dementia in diabetes mellitus: a systematic review. Lancet Neurol. 2006;5(1):64–74. | 1052 |
| 12 | Barrett JC, Clayton DG, Concannon P, et al. Genome-wide association study and meta-analysis find that over 40 loci affect risk of type 1 diabetes. Nat Genet. 2009;41(6):703–707. | 1008 |
| 13 | de Groot M, Anderson R, Freedland KE. Association of depression and diabetes complications: a meta-analysis. Psychosom Med. 2001;63(4):619–630. | 995 |
| 14 | Vestergaard P. Discrepancies in bone mineral density and fracture risk in patients with type 1 and type 2 diabetes--a meta-analysis. Osteoporos Int. 2007;18(4):427–444. | 963 |
| 15 | Boulé NG, Haddad E, Kenny GP. Effects of exercise on glycemic control and body mass in type 2 diabetes mellitus: a meta-analysis of controlled clinical trials. JAMA. 2001;286(10):1218–1227. | 960 |
| 16 | Norris SL, Lau J, Smith SJ, Schmid CH, Engelgau MM. Self-management education for adults with type 2 diabetes: a meta-analysis of the effect on glycemic control. Diabetes Care. 2002;25(7):1159–1171. | 952 |
| 17 | Selvin E, Marinopoulos S, Berkenblit G, et al. Meta-analysis: glycosylated hemoglobin and cardiovascular disease in diabetes mellitus. Ann Intern Med. 2004;141(6):421–431. | 924 |
| 18 | Huxley R, Barzi F, Woodward M. Excess risk of fatal coronary heart disease associated with diabetes in men and women: meta-analysis of 37 prospective cohort studies. BMJ. 2006;332(7533):73–78. | 885 |
| 19 | Lincoff, AM; Wolski, K; Nicholls. Pioglitazone and risk of cardiovascular events in patients with type 2 diabetes mellitus - A meta-analysis of randomized trials. JAMA. 2007;298(10):1180-1188 | 876 |
| 20 | Ray, KK; Seshasai, SRK; Wijesuriya, S. Effect of intensive control of glucose on cardiovascular outcomes and death in patients with diabetes mellitus: a meta-analysis of randomised controlled trials. Lancet. 2009;373(9677):1765-1772 | 824 |
| 21 | Preiss D, Seshasai SR, Welsh P, et al. Risk of incident diabetes with intensive-dose compared with moderate-dose statin therapy: a meta-analysis. JAMA. 2011;305(24):2556–2564. | 822 |
| 22 | Ding EL, Song Y, Malik VS, Liu S. Sex differences of endogenous sex hormones and risk of type 2 diabetes: a systematic review and meta-analysis. JAMA. 2006;295(11):1288–1299. | 800 |
| 23 | Amori RE, Lau J, Pittas AG. Efficacy and safety of incretin therapy in type 2 diabetes: systematic review and meta-analysis. JAMA. 2007;298(2):194–206. | 742 |
| 24 | Cramer JA. A systematic review of adherence with medications for diabetes. Diabetes Care. 2004;27(5):1218–1224. | 732 |
| 25 | Mezuk B, Eaton WW, Albrecht S. Depression and type 2 diabetes over the lifespan: a meta-analysis. Diabetes Care. 2008;31(12):2383–2390. | 731 |
| 26 | Cappuccio FP, D'Elia L, Strazzullo P. Quantity and quality of sleep and incidence of type 2 diabetes: a systematic review and meta-analysis. Diabetes Care. 2010;33(2):414–420. | 730 |
| 27 | Wilmot EG, Edwardson CL, Achana FA. Sedentary time in adults and the association with diabetes, cardiovascular disease and death: systematic review and meta-analysis. Diabetologia. 2012;55(11):2895–2905. | 725 |
| 28 | Huxley R, Ansary-Moghaddam A, Berrington de González A, Barzi F, Woodward M. Type-II diabetes and pancreatic cancer: a meta-analysis of 36 studies. Br J Cancer. 2005;92(11):2076–2083. | 671 |
| 29 | Jeon CY, Murray MB. Diabetes mellitus increases the risk of active tuberculosis: a systematic review of 13 observational studies. PLoS Med. 2008;5(7):e152. | 669 |
| 30 | Willi C, Bodenmann P, Ghali WA, Faris PD, Cornuz J. Active smoking and the risk of type 2 diabetes: a systematic review and meta-analysis. JAMA. 2007;298(22):2654–2664. | 667 |
| 31 | Gillies CL, Abrams KR, Lambert PC, et al. Pharmacological and lifestyle interventions to prevent or delay type 2 diabetes in people with impaired glucose tolerance: systematic review and meta-analysis. BMJ. 2007;334(7588):299. | 649 |
| 32 | Elliott WJ, Meyer PM. Incident diabetes in clinical trials of antihypertensive drugs: a network meta-analysis Lancet. 2007;369(9557):201–207. | 645 |
| 33 | Larsson SC, Orsini N, Wolk A. Diabetes mellitus and risk of colorectal cancer: a meta-analysis. J Natl Cancer Inst. 2005;97(22):1679–1687. | 631 |
| 34 | Ali S, Stone MA, Peters JL. The prevalence of co-morbid depression in adults with Type 2 diabetes: a systematic review and meta-analysis. Diabet Med. 2006;23(11):1165–1173. | 621 |
| 35 | Janghorbani M, Van Dam RM, Willett WC. Systematic review of type 1 and type 2 diabetes mellitus and risk of fracture. Am J Epidemiol. 2007;166(5):495–505. | 617 |
| 36 | Li S, Shin HJ, Ding EL, van Dam RM. Adiponectin levels and risk of type 2 diabetes: a systematic review and meta-analysis. JAMA. 2009;302(2):179–188. | 606 |
| 37 | Micha R, Wallace SK, Mozaffarian D. Red and processed meat consumption and risk of incident coronary heart disease, stroke, and diabetes mellitus: a systematic review and meta-analysis. Circulation. 2010;121(21):2271–2283. | 597 |
| 38 | Whincup PH, Kaye SJ, Owen CG, et al. Birth weight and risk of type 2 diabetes: a systematic review. JAMA. 2008;300(24):2886–2897. | 568 |
| 39 | Knol MJ, Twisk JW, Beekman AT. Depression as a risk factor for the onset of type 2 diabetes mellitus. A meta-analysis. Diabetologia. 2006;49(5):837–845. | 534 |
| 40 | Umpierre D, Ribeiro PA, Kramer CK, et al. Physical activity advice only or structured exercise training and association with HbA1c levels in type 2 diabetes: a systematic review and meta-analysis. JAMA. 2011;305(17):1790–1799. | 531 |
| 41 | DIAbetes Genetics Replication And Meta-analysis (DIAGRAM) Consortium; Asian Genetic Epidemiology Network Type 2 Diabetes (AGEN-T2D) Consortium; South Asian Type 2 Diabetes (SAT2D) Consortium; Genome-wide trans-ancestry meta-analysis provides insight into the genetic architecture of type 2 diabetes susceptibility. Nat Genet. 2014;46(3):234–244. | 529 |
| 42 | Moran LJ, Misso ML, Wild RA, Norman RJ. Impaired glucose tolerance, type 2 diabetes and metabolic syndrome in polycystic ovary syndrome: a systematic review and meta-analysis. Hum Reprod Update. 2010;16(4):347–363. | 528 |
| 43 | Browning LM, Hsieh SD, Ashwell M. A systematic review of waist-to-height ratio as a screening tool for the prediction of cardiovascular disease and diabetes: 0·5 could be a suitable global boundary value. Nutr Res Rev. 2010;23(2):247–269. | 526 |
| 44 | Larsson SC, Mantzoros CS, Wolk A. Diabetes mellitus and risk of breast cancer: a meta-analysis. Int J Cancer. 2007;121(4):856–862. | 525 |
| 45 | Decensi A, Puntoni M, Goodwin P, et al. Metformin and cancer risk in diabetic patients: a systematic review and meta-analysis. Cancer Prev Res (Phila). 2010;3(11):1451–1461. | 523 |
| 46 | Bolen S, Feldman L, Vassy J, et al. Systematic review: comparative effectiveness and safety of oral medications for type 2 diabetes mellitus. Ann Intern Med. 2007;147(6):386–399. | 515 |
| 47 | Deakin T, McShane CE, Cade JE, Williams RD. Group based training for self-management strategies in people with type 2 diabetes mellitus. Cochrane Database Syst Rev. 2005;(2):CD003417. | 498 |
| 48 | El-Serag HB, Hampel H, Javadi F. The association between diabetes and hepatocellular carcinoma: a systematic review of epidemiologic evidence. Clin Gastroenterol Hepatol. 2006;4(3):369–380. | 496 |
| 49 | Renders CM, Valk GD, Griffin SJ. Interventions to improve the management of diabetes in primary care, outpatient, and community settings: a systematic review. Diabetes Care. 2001;24(10):1821–1833. | 480 |
| 50 | Barone BB, Yeh HC, Snyder CF, et al. Long-term all-cause mortality in cancer patients with preexisting diabetes mellitus: a systematic review and meta-analysis. JAMA. 2008;300(23):2754–2764. | 474 |
| 51 | Lago RM, Singh PP, Nesto RW. Congestive heart failure and cardiovascular death in patients with prediabetes and type 2 diabetes given thiazolidinediones: a meta-analysis of randomised clinical trials. Lancet. 2007;370(9593):1129–1136. | 472 |
| 52 | Vazquez G, Duval S, Jacobs DR Jr, Silventoinen K. Comparison of body mass index, waist circumference, and waist/hip ratio in predicting incident diabetes: a meta-analysis. Epidemiol Rev. 2007;29:115–128. | 463 |
| 53 | Hahn D, Hodson EM, Fouque D. Low protein diets for non-diabetic adults with chronic kidney disease. Cochrane Database Syst Rev. 2018;10(10):CD001892. Published 2018 Oct 4. | 463 |
| 54 | Mohamed Q, Gillies MC, Wong TY. Management of diabetic retinopathy: a systematic review. JAMA. 2007;298(8):902–916. | 456 |
| 55 | Gonzalez JS, Peyrot M, McCarl LA, et al. Depression and diabetes treatment nonadherence: a meta-analysis. Diabetes Care. 2008;31(12):2398–2403. | 454 |
| 56 | Yeh GY, Eisenberg DM, Kaptchuk TJ, Phillips RS. Systematic review of herbs and dietary supplements for glycemic control in diabetes. Diabetes Care. 2003;26(4):1277–1294. | 454 |
| 57 | Patterson R, McNamara E, Tainio M, et al. Sedentary behaviour and risk of all-cause, cardiovascular and cancer mortality, and incident type 2 diabetes: a systematic review and dose response meta-analysis. Eur J Epidemiol. 2018;33(9):811–829. | 452 |
| 58 | Hanefeld M, Cagatay M, Petrowitsch T. Acarbose reduces the risk for myocardial infarction in type 2 diabetic patients: meta-analysis of seven long-term studies. Eur Heart J. 2004;25(1):10–16. | 434 |
| 59 | Fox CS, Matsushita K, Woodward M, et al. Associations of kidney disease measures with mortality and end-stage renal disease in individuals with and without diabetes: a meta-analysis. Lancet. 2012;380(9854):1662–1673. | 431 |
| 60 | de Munter JS, Hu FB, Spiegelman D. Whole grain, bran, and germ intake and risk of type 2 diabetes: a prospective cohort study and systematic review. PLoS Med. 2007;4(8):e261. | 428 |
| 61 | van Dam RM, Hu FB. Coffee consumption and risk of type 2 diabetes: a systematic review. JAMA. 2005;294(1):97–104. | 424 |
| 62 | Vasilakou D, Karagiannis T, Athanasiadou E, et al. Sodium-glucose cotransporter 2 inhibitors for type 2 diabetes: a systematic review and meta-analysis. Ann Intern Med. 2013;159(4):262–274. | 412 |
| 63 | Vanstone M, Rewegan A, Brundisini F. Patient Perspectives on Quality of Life With Uncontrolled Type 1 Diabetes Mellitus: A Systematic Review and Qualitative Meta-synthesis. Ont Health Technol Assess Ser. 2015;15(17):1–29. | 403 |
| 64 | Thomas DE, Elliott EJ, Naughton GA. Exercise for type 2 diabetes mellitus. Cochrane Database Syst Rev. 2006;(3):CD002968. Published 2006 Jul 19. | 401 |
| 65 | de Souza RJ, Mente A, Maroleanu A, et al. Intake of saturated and trans unsaturated fatty acids and risk of all cause mortality, cardiovascular disease, and type 2 diabetes: systematic review and meta-analysis of observational studies. BMJ. 2015;351:h3978. Published 2015 Aug 11. | 397 |
| 66 | Li XH, Yu FF, Zhou YH. Association between alcohol consumption and the risk of incident type 2 diabetes: a systematic review and dose-response meta-analysis. Am J Clin Nutr. 2016;103(3):818–829. | 393 |
| 67 | Montori VM, Basu A, Erwin PJ. Posttransplantation diabetes: a systematic review of the literature. Diabetes Care. 2002;25(3):583–592. | 389 |
| 68 | Snowling NJ, Hopkins WG. Effects of different modes of exercise training on glucose control and risk factors for complications in type 2 diabetic patients: a meta-analysis. Diabetes Care. 2006;29(11):2518–2527. | 387 |
| 69 | Hemmingsen B, Lund SS, Gluud C, et al. Targeting intensive glycaemic control versus targeting conventional glycaemic control for type 2 diabetes mellitus. Cochrane Database Syst Rev. 2013;(11):CD008143. | 372 |
| 70 | Cho YS, Chen CH, Hu C, et al. Meta-analysis of genome-wide association studies identifies eight new loci for type 2 diabetes in east Asians. Nat Genet. 2011;44(1):67–72. Published 2011 Dec 11. | 365 |
| 71 | Cooper C, Sommerlad A, Lyketsos CG. Modifiable predictors of dementia in mild cognitive impairment: a systematic review and meta-analysis. Am J Psychiatry. 2015;172(4):323–334. | 359 |
| 72 | Harder T, Rodekamp E, Schellong K, Dudenhausen JW, Plagemann A. Birth weight and subsequent risk of type 2 diabetes: a meta-analysis. Am J Epidemiol. 2007;165(8):849–857. | 358 |
| 73 | van de Laar FA, Lucassen PL, Akkermans RP, van de Lisdonk EH, Rutten GE, van Weel C. Alpha-glucosidase inhibitors for patients with type 2 diabetes: results from a Cochrane systematic review and meta-analysis. Diabetes Care. 2005;28(1):154–163. | 350 |
| 74 | Baker MA, Harries AD, Jeon CY, et al. The impact of diabetes on tuberculosis treatment outcomes: a systematic review. BMC Med. 2011;9:81. Published 2011 Jul 1. | 348 |
| 75 | Baliunas DO, Taylor BJ, Irving H, et al. Alcohol as a risk factor for type 2 diabetes: A systematic review and meta-analysis. Diabetes Care. 2009;32(11):2123–2132. | 347 |
| 76 | Zipitis CS, Akobeng AK. Vitamin D supplementation in early childhood and risk of type 1 diabetes: a systematic review and meta-analysis. Arch Dis Child. 2008;93(6):512–517. | 346 |
| 77 | Tricco AC, Ivers NM, Grimshaw JM, et al. Effectiveness of quality improvement strategies on the management of diabetes: a systematic review and meta-analysis. Lancet. 2012;379(9833):2252–2261. | 344 |
| 78 | Torloni MR, Betrán AP, Horta BL, et al. Prepregnancy BMI and the risk of gestational diabetes: a systematic review of the literature with meta-analysis. Obes Rev. 2009;10(2):194–203. | 342 |
| 79 | Benichou T, Pereira B, Mermillod M, et al. Heart rate variability in type 2 diabetes mellitus: A systematic review and meta-analysis. PLoS One. 2018;13(4):e0195166. Published 2018 Apr 2. | 335 |
| 80 | Nouwen A, Winkley K, Twisk J, et al. Type 2 diabetes mellitus as a risk factor for the onset of depression: a systematic review and meta-analysis. Diabetologia. 2010;53(12):2480–2486. | 331 |
| 81 | Noto H, Goto A, Tsujimoto T, Noda M. Cancer risk in diabetic patients treated with metformin: a systematic review and meta-analysis. PLoS One. 2012;7(3):e33411. | 329 |
| 82 | Agardh E, Allebeck P, Hallqvist J, Moradi T, Sidorchuk A. Type 2 diabetes incidence and socio-economic position: a systematic review and meta-analysis. Int J Epidemiol. 2011;40(3):804–818. | 328 |
| 83 | Cooper JD, Smyth DJ, Smiles AM, et al. Meta-analysis of genome-wide association study data identifies additional type 1 diabetes risk loci. Nat Genet. 2008;40(12):1399–1401. | 324 |
| 84 | Schulze MB, Schulz M, Heidemann C. Fiber and magnesium intake and incidence of type 2 diabetes: a prospective study and meta-analysis. Arch Intern Med. 2007;167(9):956–965. | 324 |
| 85 | Emdin CA, Rahimi K, Neal B. Blood pressure lowering in type 2 diabetes: a systematic review and meta-analysis. JAMA. 2015;313(6):603–615. | 323 |
| 86 | Pickup JC, Sutton AJ. Severe hypoglycaemia and glycaemic control in Type 1 diabetes: meta-analysis of multiple daily insulin injections compared with continuous subcutaneous insulin infusion. Diabet Med. 2008;25(7):765–774. | 323 |
| 87 | Roy T, Lloyd CE. Epidemiology of depression and diabetes: a systematic review. J Affect Disord. 2012;142 Suppl:S8–S21. | 317 |
| 88 | Kasper JS, Giovannucci E. A meta-analysis of diabetes mellitus and the risk of prostate cancer. Cancer Epidemiol Biomarkers Prev. 2006;15(11):2056–2062. | 316 |
| 89 | Ajala O, English P, Pinkney J. Systematic review and meta-analysis of different dietary approaches to the management of type 2 diabetes. Am J Clin Nutr. 2013;97(3):505–516. | 315 |
| 90 | Ben Q, Xu M, Ning X, et al. Diabetes mellitus and risk of pancreatic cancer: A meta-analysis of cohort studies. Eur J Cancer. 2011;47(13):1928–1937. | 315 |
| 91 | Mitri J, Muraru MD, Pittas AG. Vitamin D and type 2 diabetes: a systematic review. Eur J Clin Nutr. 2011;65(9):1005–1015. | 315 |
| 92 | Profenno LA, Porsteinsson AP, Faraone SV. Meta-analysis of Alzheimer's disease risk with obesity, diabetes, and related disorders. Biol Psychiatry. 2010;67(6):505–512. | 314 |
| 93 | Bennett CM, Guo M, Dharmage SC. HbA(1c) as a screening tool for detection of Type 2 diabetes: a systematic review [published correction appears in Diabet Med. 2007 Sep;24(9):1054]. Diabet Med. 2007;24(4):333–343. | 312 |
| 94 | Loke YK, Singh S, Furberg CD. Long-term use of thiazolidinediones and fractures in type 2 diabetes: a meta-analysis. CMAJ. 2009;180(1):32–39. | 311 |
| 95 | Cardwell CR, Stene LC, Joner G, et al. Caesarean section is associated with an increased risk of childhood-onset type 1 diabetes mellitus: a meta-analysis of observational studies. Diabetologia. 2008;51(5):726–735. | 307 |
| 96 | Pan A, Sun Q, Bernstein AM, et al. Red meat consumption and risk of type 2 diabetes: 3 cohorts of US adults and an updated meta-analysis. Am J Clin Nutr. 2011;94(4):1088–1096. | 304 |
| 97 | Friberg E, Orsini N, Mantzoros CS, Wolk A. Diabetes mellitus and risk of endometrial cancer: a meta-analysis. Diabetologia. 2007;50(7):1365–1374. | 304 |
| 98 | Stettler C, Allemann S, Jüni P, et al. Glycemic control and macrovascular disease in types 1 and 2 diabetes mellitus: Meta-analysis of randomized trials. Am Heart J. 2006;152(1):27–38. | 304 |
| 99 | Shekelle PG, Rich MW, Morton SC, et al. Efficacy of angiotensin-converting enzyme inhibitors and beta-blockers in the management of left ventricular systolic dysfunction according to race, gender, and diabetic status: a meta-analysis of major clinical trials. J Am Coll Cardiol. 2003;41(9):1529–1538. | 303 |
| 100 | Abuissa H, Jones PG, Marso SP, O'Keefe JH Jr. Angiotensin-converting enzyme inhibitors or angiotensin receptor blockers for prevention of type 2 diabetes: a meta-analysis of randomized clinical trials. J Am Coll Cardiol. 2005;46(5):821–826. | 301 |
